# Developing an integrated algorithm to support autism diagnostic decisions: Model performance and statistical fairness

**DOI:** 10.1002/jcv2.70157

**Published:** 2026-08-04

**Authors:** Aaron J. Kaat, Ashlynn Campagna, Hannah Feiner, Jeffrey Michael Grauzer, Amanda Nicole Hobson, Maranda Jones, Jordan Paige Lee, Laura Jean Sudec, Megan York Roberts

**Affiliations:** ^1^ Department of Medical Social Sciences Northwestern University Feinberg School of Medicine Chicago Illinois USA; ^2^ Institute for Innovations in Developmental Sciences Northwestern University Chicago Illinois USA; ^3^ Communication Sciences and Disorders Northwestern University School of Communication Evanston Illinois USA

**Keywords:** autism, elastic net regression, machine learning, TELE‐PEDS‐ASD, toddler autism symptom interview

## Abstract

**Background:**

Best practices in diagnosing autism spectrum disorder require an expert diagnostician to integrate multiple sources of information, including direct observations, interviews, and rating scales completed by knowledgeable informants. Machine learning methods are well‐positioned to integrate disparate information sources to improve classification. This study sought to evaluate whether a machine learning algorithm could support appropriately weighting multiple sources of diagnostically relevant information.

**Methods:**

Telehealth diagnostic assessments were conducted for 639 toddlers (age mean = 30.4, SD = 4.3 months; 29.7% female) already receiving early intervention (EI). The Toddler Autism Symptom Interview (TASI) and the TELE‐ASD‐PEDS (TAP) were completed during separate visits. Each child's caregivers and usual EI providers asynchronously completed rating scales of autistic characteristics developed for this study. Calibration and validation data subsets were created using a 2:1 split. The optimal algorithm was developed using elastic net regularized regression in the calibration dataset, which was then evaluated in the validation dataset. Statistical fairness criteria evaluated whether the proposed algorithm functioned similarly across multiple protected group statuses.

**Results:**

The prevalence of autism in the sample was 80.4%. The algorithm developed in the calibration dataset included both the caregiver‐ and EI provider‐completed rating scales, four items from the TASI, and six items from the TAP. Model performance was high in both the calibration and validation samples (sensitivity >0.90; specificity >0.75; kappa >0.60), but statistical fairness criteria varied. False positives were relatively rare, but were more common in advantaged groups, which may suggest systematic under‐classification by the algorithm in minoritized groups.

**Conclusion:**

Diagnostic practices for autism require integrating multiple sources of information. Machine learning can support diagnosticians in this effort, as demonstrated here utilizing multiple rating scales, the TASI, and the TAP. However, there is a risk for bias when using machine learning and as such, no algorithm should replace expert clinical judgment.

## Key Points

**What's known?**

- Machine learning techniques are becoming more common in autism research. Classification models in particular may be beneficial for integrating multiple sources of information in the diagnostic context. As machine learning methods develop, there has been an increasing demand for ensuring fairness of these algorithms, which also must translate into applied research in autism.

**What's new?**

- This study utilized data from over 600 toddlers receiving early intervention (EI) services suspected of being autistic (80% of whom received diagnoses). They were evaluated using rating scales, a structured interview, and a standardized observation conducted over telehealth. Regularized regression techniques were utilized to develop an algorithm that integrates these multiple sources of information.
- The algorithm was highly accurate in both the calibration and validation subsamples. It differentially weighted the rating scales and specific items from the interview and observation measures. Given that most of the sample received an autism diagnosis by the expert diagnostic team, the sensitivity of the algorithm was excellent, but specificity was also very good.
- However, statistical fairness criteria suggested that the algorithm did not function similarly by protected group status. Algorithmic misclassifications for toddlers from advantaged groups (i.e., children assigned male at birth, White children, children whose caregivers expressed prior concern, and/or who were not exhibiting cognitive delays) were made in both directions, whereas false negatives were more likely to occur than false positives among toddlers from minoritized groups. This suggests a lack of fairness in the algorithm.

**What's relevant?**

- The findings of this study are relevant to future researchers interested in applying machine learning techniques. Algorithms are regularly being developed, but less attention has been paid to whether they are fair for all individuals suspected of being autistic. These findings are also relevant to clinicians and diagnosticians. This study demonstrates that machine learning algorithms can support integrating multiple sources of information to improve diagnostic decision‐making, but it also demonstrates that an algorithm is not a replacement for an expert diagnostic team meeting consensus on best clinical estimates.

## INTRODUCTION

Autism spectrum disorder is a behaviorally‐defined condition that can be reliably diagnosed by 24 months of age (Dawson et al., 2023; Kleinman et al., 2008; Lord et al., 2006; Pierce et al., 2019). Despite this, the median age of diagnosis remains approximately 4 years old, with variability across the country from around 3 to over 5‐1/2 years (Shaw et al., 2025). In some cases, autistic toddlers are enrolled in services for developmental delays or co‐occurring conditions prior to formally receiving their diagnosis. Indeed, a recent study found that implementing specific screening for autism within EI resulted in increased identification of autistic toddlers already receiving services, which was most pronounced for minoritized families and thereby reduced diagnostic disparities in the EI system (Sheldrick et al., 2022). Screening for autism is recommended by the US Preventive Services Task Force in order to improve access to EI (Siu et al., 2016), but conducting screening within the EI context would allow access to specialized services (both within EI and supplemental to it).

Though there is some variability in the diagnostic process, best practices recommend utilization of a multidisciplinary (or interdisciplinary) team using standardized assessment tools with appropriate validity evidence, recognizing that the output of a standardized tool is not the same as a diagnosis—a diagnosis is based on a skilled diagnostician integrating all relevant data to determine case status (Bishop & Lord, 2023). Minimally, the diagnostic process should include a direct assessment, an interview with a knowledgeable informant, an assessment of functional strengths and weaknesses, and a determination regarding the amount of supports an individual would require. Integration of all these information sources is a necessary component of any diagnostic evaluation, yet is often insufficiently addressed in training and guidance.

Machine learning techniques provide an opportunity to integrate multiple disparate but correlated features in a classification problem—which is exactly what diagnosticians are expected to be able to do. Classification algorithms can accelerate diagnostic efforts by identifying the most salient diagnostic features and reducing redundancy while maintaining high degrees of accuracy. Further, they can provide a predicted probability of a diagnosis that can support a diagnostician in their decision‐making process. Already machine learning algorithms are being used for diagnostic purposes, both to improve diagnostic measures (Wagner et al., 2025) and for data that are not part of routine diagnostic evaluations, such as eye tracking patterns or bodily movements (Jones et al., 2023; Simeoli et al., 2024). There are also examples of using item‐level data from autism diagnostic instruments to try to improve accuracy of the measures in the presence of co‐occurring conditions (e.g., Schulte‐Rüther et al., 2023). Aggregating data across measures that are routinely collected in the diagnostic process, however, remains an important area of growth.

As more studies utilize machine learning algorithms, not only for autism diagnostic purpose but across healthcare contexts, there are increasing calls for ensuring that the algorithms treat people fairly and equitably (Gichoya et al., 2021; Rajkomar et al., 2018). Test fairness is not a new concept; it has long been a consideration within validity theory and standards for test development under the guise of mitigating test bias (American Educational Research et al., 2014; Cleary, 1968; Messick, 1995; Thissen et al., 1986). In the context of diagnostic decisions, this becomes a concern of differential predictive validity, which has been defined as when “a test is biased for members of a subgroup of the population in the prediction of a criterion for which the test was designed, consistent nonzero errors of predication are made for members of the subgroup” (Cleary, 1968). With the outgrowth in machine learning algorithms, formal fairness criteria have been proposed to address this same problem (Mitchell et al., 2021; Rajkomar et al., 2018). Unfortunately, many of the criteria used to evaluate algorithmic fairness are in conflict with each other: to be fair in one dimension may result in inequities by another statistical definition (Beigang, 2022, 2023). Nonetheless, one can use multiple fairness criteria to create a cogent validity argument for or against fairness by protected group status (Mitchell et al., 2021; Rajkomar et al., 2018).

The purpose of this study was first to improve diagnostic practices by utilizing machine learning algorithms to integrate multiple sources of information in the diagnostic process and second to evaluate whether any proposed algorithm is statistically fair for relevant subpopulations within the autistic sample. This algorithm is not intended to replace clinical judgement—just like the score from a diagnostic instrument is not the same as a clinical diagnosis, the classification from a machine learning algorithm is meant to support the judgment of the diagnostician, not replace it. Nonetheless, the assessment battery utilized herein is proposed as a more accessible evaluation than traditional diagnostic evaluations, which may mean that the diagnosticians utilizing it could benefit from additional support for integrating the data sources. We intend to make the algorithm available as a spreadsheet for clinicians interested in utilizing this battery for diagnostic purposes, where it could provide them with a probability of a diagnosis and a likely classification assuming the samples are similar to this one.

## MATERIALS AND METHODS

### Participants

This study was ancillary to a double masked diagnostic accuracy study evaluating whether EI teams trained to conduct autism evaluations were comparable to an expert diagnostic team (Roberts, under review). As such, all participants were evaluated by an EI diagnostic team, but for the purposes of these analyses, final best clinical estimate diagnosis was determined based on the classifications from the expert diagnostic team, which was composed of a licensed clinical psychologist, an EI‐credentialed Developmental Therapist (DT, which are EI therapists who provide general special education services within the Illinois EI system), and a licensed Speech‐Language Pathologist (SLP), who had over 15 years of experience in multidisciplinary autism diagnostic settings. Parents or legal guardians provided written consent and all study procedures were approved by the institutional review board.

Inclusion criteria for the primary study were children (a) currently enrolled in the Illinois EI Program (eligibility for services varied across children but were most often due to speech or language delays or due to global developmental concerns), (b) between the ages of 15–36 months of age, and (c) who understood and responded to either English or Spanish. Exclusion criteria focused on co‐occurring medical conditions, specifically blindness, cerebral palsy, Down syndrome, genetic syndrome or chromosome deletion, hearing loss, seizures, stroke, and/or traumatic brain injury. Participants who reported using a feeding tube or GI tube were also excluded. The expert team also reviewed any other diagnoses that the potential participant reported to determine if it would be better for them to have a physician as part of the diagnostic team, if other diagnoses with reported. Additionally, the child's legal guardian had to (a) speak English or Spanish without an interpreter and (b) agree to a video‐recorded virtual autism evaluation. These inclusion and exclusion criteria were selected because children with co‐occurring medical conditions that may impact diagnostic assessments are better suited for an evaluation with a developmental and behavioral pediatrician and thus are not appropriate candidates for the alternate diagnostic pathway evaluated in the primary study. Further, as related to the current study, we intend for the algorithm to detect autism caseness, not other co‐occurring conditions, and therefore excluding these cases should improve the accuracy of any algorithm.

### Measures

The Toddler Autism Symptom Interview (TASI; Coulter et al., 2021) is a 37‐item semi‐structured interview designed for caregivers of children between 12 and 36 months of age. Items are nested within one of the criteria from the Diagnostic and Statistical Manual fifth Edition (DSM‐5) for autism spectrum disorder, such that each criterion is represented by several interview items. Additionally, there is a table for eliciting information about common autistic sensory experiences. Item scores are then summed within DSM‐5 criteria. The overall score ranges from 0 to 33, with different ranges by diagnostic criterion. Any total score greater than or equal to seven is indicative of an increased likelihood of autism.

The TELE‐ASD‐PEDS (TAP; Wagner et al., 2021) is a synchronous, virtually‐administered structured observation appropriate for toddlers between 14 and 36 months of age. A clinician coaches the caregiver, who mediates and implements eight activities designed to elicit social behaviors. Immediately after this observation, clinicians rate seven diagnostically‐salient behaviors on a three‐point scale (1, characteristic of autism not present; 2, possible atypical behavior; 3, behaviors characteristic of autism clearly present). Scores range from 0 to 21, and any score greater than or equal to 12 is indicative of an increased likelihood of autism.

Caregivers also completed the Developmental Profile—fourth Edition (DP4; Alpern, 2020). The DP4 is a survey‐based assessment of five developmental domains: physical, adaptive behavior, cognitive, social‐emotional, and language. Items are rated yes/no in each domain. The DP4 provides age‐based standard scores (mean = 100, sd = 15) and growth scale values, which are person‐ability scores that are appropriate for outcome assessment (Farmer et al., 2020).

Caregivers completed other measures as well, including a checklist developed for this study (completed by one or multiple caregivers) of autism characteristics, a medical history form, and other sociodemographic information including whether or not they had a concern of autism prior to this evaluation referral. The survey measure of autism characteristics was developed by the study team to use neurodiversity‐affirming language and operationalized DSM criteria in 18 yes/no questions. The survey measure was designed for multiple types of informants, including but not limited to caregivers. As such, each child's current EI team was also invited to complete a version of the autism characteristic checklist with minor language modified to reflect non‐caregiver informants. For some toddlers, no providers on their personal EI team completed the scale, while others had up to three EI informants.

### Procedures

DTs and SLPs with at least 1 year of experience who had an active Illinois EI credential were eligible to serve on an EI Diagnostic Team. Early intervention therapists were randomly assigned to a team, such that each team had one SLP and one DT. There were 51 unique EI therapist teams, which were trained in cohorts by the research team using a multi‐method, case‐based learning approach with structured discussion (Mazurek et al., 2019; Nowell et al., 2020). This training included seven required self‐guided modules covering topics such as culturally responsive and identity‐affirming practices, DSM‐5 diagnostic criteria (APA, 2013), administering the caregiver interview, administering the child observation, making a best clinical estimate, and effectively communicating the diagnosis to families. After completing the modules, therapists watched all elements of the autism evaluation process, scoring each of the measures and providing their best clinical estimate for the case. Total training time was approximately 20 hours. Each EI team provided autism evaluations for approximately 14 children over the course of 16 weeks.

The evaluation consisted of three appointments with the child and/or their caregivers. Prior to the synchronous virtual visits, caregivers completed the sociodemographic and clinical history information, the DP4, and the checklist of autism characteristics. Diagnostic instruments included the TASI and the TAP, which were administered during two separate visits.

Following each child's second appointment, the EI diagnostic team individually rated the child on the autism diagnostic criteria specified in the DSM‐5 (APA, 2013) and then met to collaboratively determine an appropriate diagnosis for the child. Separately, the expert diagnostic team reviewed the video recordings from the TAP and TASI and the survey measures and determined the best clinical estimate from all available information. Diagnoses were made based on the DSM‐5, which requires meeting criteria in both the social communication and restricted or repetitive behaviors and interests domains (APA, 2013). The EI team then met with the family for the third visit, at which point they reviewed recommendations for intervention (regardless of diagnostic outcome) and the consensus diagnosis of autism. The comparison between EI diagnosis and expert diagnosis is provided elsewhere (Roberts, under review). For the current study, only the expert diagnosis was utilized for classification modeling.

### Data analysis

In order to combine data from multiple informants, we utilized a stage‐based method to include relevant predictors. We split the full dataset into calibration and validation subsamples using a 2:1 split. The split was stratified by sex assigned at birth, race/ethnicity, and best clinical estimate (autistic vs. other) to maintain approximate equal proportions in both subsets. Within the calibration dataset, we utilized 10‐fold cross‐validation to ensure the robustness of our results. Models were initially built in the calibration dataset, with two thresholds determined based on examining putative values that would separately maximize negative and positive predictive values (Roberts et al., 2018). The predictive model was then applied to the validation dataset with caseness determined based on whether the expected value was below the lowest threshold (likely non‐autistic), between thresholds (needs further evaluation), or above the highest threshold (likely autistic). For classification purposes, likely non‐autistic and needs further evaluation classifications were collapsed so that the model performance would be based on likely autistic versus any other classification.

First, we wanted to reduce the number of predictors in a gated fashion in the calibration dataset. For the caregiver‐ and provider surveys of autism characteristics, we conducted an elastic net regression analysis utilizing only the dichotomous items within those measures to get an expected probability of an ASD diagnosis. Elastic net is a machine learning type that combines both lasso and ridge regularization to generalized linear models; it is useful for feature selection in the presence of a large number of potentially correlated predictor variables (Zou & Hastie, 2005). Then we entered all TAP and TASI items and the expected probabilities from the caregiver survey and a randomly‐selected provider survey (if the participant had more than one provider survey) into a second elastic net regression model. Cross‐validation was utilized to get the optimal lambda value for identifying the most predictive variables without including irrelevant variables.

We evaluated model performance using several statistics from the classification and machine learning literature. Traditional statistical indices, including sensitivity, specificity, positive and negative predictive values (PPV and NPV, respectively), and Cohen's kappa were calculated. From these confusion matrices, we also calculated fairness criteria by sex assigned at birth (male/female), primary racial or ethnic identity (White, Black, or Latine—based off the largest sample sizes represented in our data), and cognitive status from the DP4 Cognition age‐based normative score (above/below 70). Then the following fairness criteria (Mitchell et al., 2021; Rajkomar et al., 2018) from the machine learning literature were calculated:

‐ Equal accuracy. This criterion quantifies the maximum difference between the proportion of correctly‐classified cases across subgroups. Lower values are better, ideally below a 10% difference.
‐ Equalized odds. This criterion conditions the fairness comparison on the results of the screening test; it calculates the maximum difference between groups on sensitivity and the maximum difference on specificity, and whichever difference is greater is reported. In this way, it penalizes both differences in the odds of being a case and the odds of not being a case. Lower values are better, ideally below 10%.
‐ Positive predictive value parity. PPV parity conditions the fairness comparison on the outcome (true state) of the individual; it is quantified based on the ratio of the smallest to the largest PPV within the subgroups. For this index, higher values are better, ideally greater than 80%.
‐ Negative predictive value parity. This is similar to the PPV parity but with NPV values. Higher ratios are preferred for this criterion as well.
‐ Treatment equality. This criterion is the maximum difference between the within‐group ratio of false positives to false negatives. Lower values are better for this criterion, but standards are less well defined, given that this is a difference in ratios.

All of these fairness criteria were reported, insofar as the statistical formulations of the equalities conflict with each other under most real‐world circumstances (Beigang, 2022, 2023). Therefore, we took a holistic approach to evaluating the algorithm, to understand for whom and under what circumstances the model performs as expected.

Further, we applied these fairness criteria to the original measure classifications from the TAP and TASI. Although sensitivity and specificity and other evidence for their predictive validity is reported elsewhere (Coulter et al., 2021; Wagner et al., 2021), for comparability we used the classification rules from the original measures to determine if unfairness in the algorithm was also present in the original measures and vice versa.

## RESULTS

There were 639 cases included in the analysis. This resulted in 426 cases in the calibration subsample and 213 cases in the validation subsample, with 80% of the sample receiving an autism diagnosis. The sample was primarily male, which is consistent both with referrals for an autism evaluation in this age range and for the demographics of EI services. The proportion of Black and Latine families was larger than typical for research samples, which reflects the diversity of the EI context. Participant demographics are provided in Table 1.

**TABLE 1:** Participant demographics.

	Full sample (*n* = 639)	Calibration (*n* = 426)	Validation (*n* = 213)
Age (months), mean (SD)	30.4 (4.3)	30.4 (4.2)	30.5 (4.5)
Female, *n* (%)	190 (29.7%)	126 (29.6%)	64 (30.0%)
Race/Ethnicity, *n* (%)			
White	204 (31.9%)	143 (33.6%)	61 (28.6%)
Black	191 (29.9%)	127 (29.8%)	64 (30.0%)
Hispanic	93 (14.6%)	55 (12.9%)	38 (17.8%)
Asian	20 (3.1%)	13 (3.1%)	7 (3.3%)
American Indian or Alaska native or native Hawaiian or other pacific Islander	8 (1.3%)	5 (1.2%)	3 (1.4%)
Multiple races	63 (9.9%)	42 (9.9%)	21 (9.9%)
Prefer not to answer	60 (9.4%)	41 (9.6%)	19 (8.9%)
Languages spoken other than English, *n* (%)			
Spanish	139 (21.8%)	89 (20.9%)	50 (23.5%)
Other languages	67 (10.5%)	45 (10.6%)	22 (10.3%)
Geographic region, *n* (%)			
Urban	535 (83.7%)	355 (83.3%)	180 (84.5%)
Rural	104 (16.3%)	71 (16.7%)	33 (15.5%)
Receiving medicaid, *n* (%)	231 (36.2%)	145 (34.0%)	86 (40.4%)
Age of first concern (months), mean (SD)	13.5 (5.9)	13.7 (5.8)	12.9 (6.0)
Age of EI enrollment (months), mean (SD)	23.5 (5.4)	23.4 (5.5)	23.5 (5.3)
Percent delay extracted from early intervention evaluation records, mean (SD)			
Adaptive development	31.7 (16.4)	30.6 (16.2)	34.0 (16.7)
Cognitive development	40.5 (17.7)	38.8 (17.4)	43.7 (17.9)
Communication development	52.4 (17.1)	50.5 (17.9)	56.6 (14.6)
Physical development	25.6 (16.1)	25.4 (15.8)	25.9 (17.2)
Social‐emotional	39.4 (18.2)	38.2 (18.0)	42.0 (18.4)
Developmental Profile‐4 scores, mean (SD)			
Physical	81.4 (20.8)	81.1 (20.7)	81.9 (21.2)
Adaptive behavior	73.5 (17.0)	73.5 (16.7)	73.6 (17.6)
Cognitive	70.7 (20.1)	71.4 (19.7)	69.4 (20.9)
Communication	60.4 (19.7)	60.5 (19.2)	60.2 (20.8)
Social‐emotional	62.4 (17.7)	62.4 (17.2)	62.5 (18.6)
Reference diagnosis of autism	514 (80.4%)	342 (80.3%)	172 (80.8%)

In the calibration sample, initial models for the within‐survey analyses suggested unexpected results. Specifically, the item reflecting the “rituals and sameness” criterion was not selected for some informants (which would not be problematic), or it was selected but in the opposite, unexpected direction. That is, a child who is already receiving EI and who has ritualistic behavior was *less likely* to receive an autism classification in this sample. Given that this is inconsistent with autism diagnostic criteria, this item was dropped from consideration for all informants. After removing that item, there were 10 items that were chosen by the elastic net model for caregivers. The clinician informant surveys varied, with 10 items for occupational therapists, 12 items for SLPs, and 13 items for DTs. All informants consistently utilized items related to directing attention, following a point, showing items, responding to name, pretend play, and instrumental gestures. Other survey items were relevant for only some of the informants. Coefficients and corresponding odds ratios for each item are shown in Supporting Information S1 Table S1.

The predicted probabilities of an autism diagnosis from the informant surveys were then aggregated with the TAP and TASI items. This resulted in 16 potential items in the primary algorithm. These items reflect a range of scaling (e.g., probabilities from the caregiver and informant surveys varied from 0 to 1; the score ranges for TASI and TAP are described above). Similar to the informant survey analyses, the item related to ritualistic and sameness behavior on the TASI was also in the opposite direction than the expectation with diagnostic criteria. As such, it was removed from consideration. Table 2 shows the parameter estimates from the elastic net machine learning model in the calibration dataset with the final item availability. As the variables are on different scales, direct comparisons of the magnitude of the coefficients are not possible. Nonetheless, it is informative which items were *not* included in the algorithm—specifically, unusual vocalizations (TAP) and deficits in social relationships and restricted interests (TASI).

**TABLE 2:** Model coefficients from the calibration subsample.

Measure	Predictor	Score range	Coefficient
Model Intercept			−7.67
TAP	Socially directed speech/sounds	1–3	0.90
	Eye contact	1–3	0.60
	Unusual vocalizations	1–3	^a^
	Unusual or repetitive play	1–3	0.17
	Unusual or repetitive body movements	1–3	0.04
	Combines gestures, eye contact, and speech/vocalization	1–3	0.47
	Unusual sensory exploration or reaction	1–3	0.28
TASI	A1 total	0–9	0.04
	A2 total	0–6	0.02
	A3 total	0–6	^a^
	B1 total	0–8	0.05
	B3 total	0–2	^a^
	B4 total	0–3	0.03
Caregiver survey	Expected probability	0–1	1.44
Informant survey	Expected probability	0–1	3.58

^a^ Indicates not selected in cross‐validated elastic net model.

This model performed relatively well in the calibration data subsample, as expected, with only minor decrement in performance when applied to the holdout validation subsample. Given that the prevalence of autism was high in this sample, specificity and NPV were the lowest model performance indices at almost any potential threshold. Youden's J recommended a single threshold of 0.78. We considered potential multiple thresholds from a scientific perspective in 0.05 increments. That information is provided in Supporting Information S1 Table S2. The final choice was a lower threshold of 0.50 and an upper threshold of 0.75. The model performance is described in Table 3.

**TABLE 3:** Model performance in the calibration and validation subsamples.

Metric	Calibration subsample		Validation subsample	
	Lower threshold	Upper threshold	Lower threshold	Upper threshold
Accuracy	0.94	0.90	0.95	0.89
Sensitivity	0.98	0.90	0.98	0.90
Specificity	0.80	0.89	0.78	0.84
PPV	0.95	0.97	0.96	0.97
NPV	0.90	0.69	0.86	0.60
Kappa	0.81	0.72	0.79	0.64

Using the upper threshold to indicate caseness, we evaluated the statistical fairness of the threshold across protected characteristics, including sex assigned at birth, race/ethnicity, prior parental concern, and cognitive status. As noted above (Beigang, 2022, 2023; Mitchell et al., 2021), these criteria are in statistical conflict with each other under most real‐world circumstances. Therefore, we use a holistic approach to evaluate the criteria presented in Table 4 to evaluate overall fairness.

**TABLE 4:** Fairness criteria.

	Characteristic	Accuracy difference	Equalized odds	PPV parity	NPV parity	Treatment equality
Proposed integrated algorithm	Sex	0.076	0.250	0.063	0.155	0.438
	Race—black versus white	0.034	0.100	0.038	0.055	0.333
	Race—other versus white	0.143	0.177	0.046	0.180	0.333
	Non‐Hispanic versus Hispanic	0.089	0.113	0.045	0.154	0.000
	Parental prior concern	0.022	0.164	0.009	0.129	0.233
	DP4 cognition	0.176	0.200	0.146	0.205	0.500
TAP	Sex	0.015	0.160	0.007	0.069	2.333
	Race—black versus white	0.011	0.125	0.033	0.471	7.750
	Race—other versus white	0.042	0.333	0.068	0.067	8.000
	Non‐Hispanic versus Hispanic	0.052	0.200	0.058	0.018	3.000
	Parental prior concern	0.017	0.271	0.033	0.429	^a^
	DP4 cognition	0.156	0.244	0.126	0.286	^a^
TASI	Sex	0.011	0.017	0.024	0.133	3.750
	Race—black versus white	0.054	0.028	0.062	0.167	7.000
	Race—other versus white	0.024	0.556	0.101	0.208	12.667
	Non‐Hispanic versus Hispanic	0.038	0.250	0.067	0.286	^a^
	Parental prior concern	0.041	0.405	0.041	0.229	2.500
	DP4 cognition	0.162	0.462	0.143	^a^	^a^

^a^ Indicates undefined values due to zero in a cell count.

The accuracy difference was acceptable for most protected characteristics, but there was inequity between groups when comparing the Other racial identity to the White racial identity, and when comparing individuals with different cognitive abilities on the DP4. The Other racial group was significantly smaller, so misclassification rates negatively affected these comparisons. For the DP4, there were very few cases of non‐autistic classifications in the lower cognitive abilities group, and accuracy differences are greatly affected by prevalence rates which may explain this pattern.

The equalized odds criterion evaluates both the sensitivity and the specificity of the proposed integrated algorithm. For all protected characteristics, this criterion suggested that the algorithm was not fair. This was driven almost exclusively by inequity in the specificity of the algorithm across groups. False positives were relatively rare in any group (indeed, there were no false positives for females or individuals with cognitive delays on the DP4, and only one false positive for Black children and for children whose caregivers did not have prior concerns of autism). Because of this, any differences by subgroup greatly affected the specificity.

PPV and NPV parity are similar to the equalized odds criterion, but condition on the best clinical estimate as opposed to the test classification. PPV parity was largely supported except by cognitive status on the DP4, where substantially more children without delays had false positive results compared to the children with delays (for whom there were no false positives). NPV parity was not supported except between Black and White racial identities. The lack of NPV parity was driven largely by unequal false negatives rates, with more false negatives for males, Other racial identities (compared to White), and children with cognitive delays on the DP4.

Treatment equality—the difference in the ratios of false positives to false negatives—is the final fairness criterion considered herein. Although specific thresholds of fairness are not widely agreed upon, poor treatment equality was noted for all comparisons except by Hispanic/Latine ethnic identity. False positives were relatively rare, while false negatives differentially affected the groups, as noted in other fairness criteria above. In all groups, false positives were more likely in children with advantaged membership (e.g., males, White children, children whose caregivers expressed prior concern, and who were not exhibiting cognitive delays on the DP4), as opposed to the minoritized protected group characteristic. This may suggest underdiagnosis in minoritized groups.

As a follow‐up analysis, we also evaluated the fairness criteria on the classification thresholds from the original measures. Although this is not typically conducted on diagnostic measures, for comparability with the proposed machine learning algorithm that integrates all of the sources of information, we chose to add these analyses. Unlike the integrated algorithm, children were more likely to be classified as autistic when they were not (i.e., false positives). This resulted in undefined fairness criteria for some comparisons when no false negatives occurred in the groups. Although the errors are in the opposite direction (overdiagnosis as opposed to underdiagnosis), the fairness criteria look similar—that is, the accuracy difference and the PPV parity are broadly fair, while the equalized odds and NPV parity are broadly unfair, and the treatment equality criterion found large differences in the ratios of false positives to false negatives per group. This suggests that the proposed integrated algorithm did not introduce any biases into the diagnostic process, but that these biases existed within the classifications on the original measure and were carried forward into the machine learning algorithm.

## DISCUSSION

Integrating multiple sources of information to form a best clinical estimate is the gold standard for autism diagnosis (Bishop & Lord, 2023). As diagnostic practices move from in‐person to virtual settings, including multiple structured diagnostic measures (Coulter et al., 2021; Wagner et al., 2021), the ability to integrate multiple sources of information becomes even more important. This study utilized machine learning methods and fairness criteria to support diagnostic decisions based on an integrated algorithm that would be equitable across groups.

We developed an informant survey with comparable content for both caregivers and children's usual EI care teams (SLPs, DTs, and OTs). Machine learning suggested that the items required informant‐specific scoring, but that the expected probability of diagnosis across the EI care team informants was comparable enough for combining. The probabilities, then, from the caregiver and from one member of the usual EI care team were aggregated with a direct assessment of the child's behavior (i.e., the TAP; Wagner et al., 2021) and a semi‐structured caregiver interview (i.e., the TASI; Coulter et al., 2021). Finally, we utilized the machine learning algorithms to integrate these data sources to support clinicians in determining a best clinical estimate.

The machine learning algorithm had very high accuracy in all groups. However, statistical fairness criteria suggested that the algorithm was not fair compared to the trained expert diagnostic team. On the one hand, the algorithm made more errors in the advantaged groups (i.e., males, White children, children whose caregivers expressed prior concern, and who were not exhibiting cognitive delays on the DP4), but on the other hand, it made systematic errors in the minoritized groups. False positives were far less likely in the minoritized protected group characteristics, which suggests systematic underdiagnosis could occur in these groups if one relied only on the algorithm. Follow‐up analyses suggested that the thresholds for caseness of the TASI and the TAP were not statistically fair across the considered protected characteristics. However, they were more likely to overclassify children—that is, overdiagnosis. Together, these findings reiterate the need for a trained diagnostician to review and interpret both the test and the algorithm classifications, and interpret them in‐context, when determining a best clinical estimate, as was done here. Nonetheless, accuracy was very high in all groups and for all methods, likely given the high prevalence of autism within this EI sample (which is consistent with other research on screening for autism in EI; Sheldrick et al., 2022).

One objection to our approach may be that some features were excluded from the final model which may lead some to question whether all items need to be administered or all prompts conducted. For example, the TASI B3 criterion (restricted interests and insistence on sameness) was not chosen in the cross‐validated model and excluded from consideration (c.f., Table 2). It would be a faster interview if the questions related to B3 were not administered. However, the TASI was administered as per its development and standard protocol, and the scoring was based on the holistic impression across the entire interview. Similarly, the TAP item on vocalizations was not utilized, but the impressions and scoring were based on the full context of the assessment and not isolated portions. Caregivers and EI professionals also had the full context of the survey without knowing which items would contribute to their score. This context is a way to frame the interpretation of the child's behavior. This diagnostic pathway is already abbreviated compared to tertiary care clinic settings; to reduce it further by eliminating portions of the test should not be considered. Best practices for a diagnostic evaluation require integration of caregiver report, direct assessment, and evaluation of language and cognitive status (Bishop & Lord, 2023). Indeed, a more thorough evaluation of language or cognition could have been considered herein, as opposed to using the DP4; this accommodation was made because the children were referred from EI, where they should have received more in‐depth testing already. Therefore, researchers and clinicians interested in utilizing the integrated algorithm we propose should be prepared to administer the surveys, TASI, and TAP in their entirety. Not only is this necessary to replicate our methods and for a complete picture of the child's functioning, but it also likely directly contributed to the satisfaction with the diagnostic process that the families reported in the study (Roberts, under review).

Nonetheless, this study is not without its limitations. First and foremost, the eligible patient population consisted of families already enrolled in EI who did not have any major medical or genetic complications, as these families are better assessed in a diagnostic setting that would include medical professionals. The prevalence of autism is higher in an EI setting than in unselected samples of toddlers, and as such, criteria that rely on the prevalence rates of a condition (like PPV and NPV) may be different in other populations. Second, because the families were already enrolled in EI, they received previous language and cognitive testing. Telehealth assessments for these skills, particularly in the toddler age range, are yet to be developed. In a different population where previous testing would not be available, a comprehensive evaluation likely would need to include individualized assessment of language and cognition rather than simply using the caregiver‐completed DP4, as was done here, potentially limiting generalizability beyond the EI context.

## CONCLUSION

Machine learning methods hold great promise for improving diagnostic practices. As high‐quality datasets become available for training these models, it is likely that more algorithms will be proposed. This study demonstrated how a classification algorithm can integrate multiple sources of information to support diagnostic decisions. The proposed algorithm largely worked well. However, it systematically underdiagnosed children from marginalized groups (noting that the original diagnostic measures systematically overdiagnosed children), which failed some statistical fairness tests likely due to the high prevalence of autism in this sample. Relying exclusively on an algorithm—or any classification from a diagnostic tool—is not best practice. Practitioners need to be aware that these biases exist and that clinical judgment is still required to determine the best clinical estimate for any individual child. More research is necessary to evaluate the consequences of these inequities. As more machine learning algorithms are proposed to support autism diagnostic evaluations, the need to evaluate statistical fairness criteria should be emphasized.

## AUTHOR CONTRIBUTIONS

**Aaron J. Kaat**: Conceptualization; investigation; funding acquisition; writing—original draft; methodology; formal analysis; project administration; supervision. **Ashlynn Campagna**: Writing—review and editing; investigation. **Hannah Feiner**: Investigation; writing—review and editing; project administration. **Jeffrey Michael Grauzer**: Writing—review and editing; formal analysis; software; data curation. **Amanda Nicole Hobson**: Investigation; writing—review and editing. **Maranda Jones.** Investigation; writing—review and editing; project administration; validation. **Jordan Paige Lee**: Investigation; writing—review and editing; project administration; resources. **Laura Jean Sudec**: Investigation; writing—review and editing; project administration; data curation; resources. **Megan York Roberts**: Conceptualization; investigation; funding acquisition; writing—review and editing; methodology; project administration; supervision; resources.

## CONFLICT OF INTEREST STATEMENT

The authors declare no conflicts of interest.

## ETHICAL CONSIDERATIONS

Informed consent and parental permission were obtained from parents or legal guardians. All study procedures were approved by the Northwestern University institutional review board under study number STU00217310 initially approved on June 15, 2022.

## Supporting information

Supporting Information S1

## Data Availability

The data that support the findings of this study are available from the corresponding author upon reasonable request.
