# Supplementary material for: Developing an integrated algorithm to support autism diagnostic decisions: Model performance and statistical fairness
Source: JCPP Adv. 2026 Aug 4:e70157. Online ahead of print. doi: 10.1002/jcv2.70157 (PMC13435154; doi:10.1002/jcv2.70157)
Supplement: Supplementary file 1 [file JCV2-9999-e70157-s001.docx]

**Developing an Integrated Algorithm to Support Autism Diagnostic Decisions: Model performance and statistical fairness**

**Supporting Information**

**Table S1: Elastic Net Classification Models for the Informant Surveys**

| Abbreviated Item | Caregiver Survey | SLP Survey | DT Survey | OT Survey |
| --- | --- | --- | --- | --- |
| (Model Intercept) | 1.81 | 1.16 | 1.16 | 0.88 |
| Points to objects | -0.64 | -0.15 | -0.44 | † |
| Shows objects | -0.39 | -0.03 | -0.39 | -0.31 |
| Follows your point | -0.36 | -0.60 | -0.11 | -0.22 |
| Responds to name | -0.31 | -0.21 | -0.18 | -0.05 |
| Directs attention to something they noticed | -0.19 | -0.80 | -0.20 | -0.34 |
| Hand as a tool (instrumental gesture) | 0.17 | 0.84 | 0.37 | 0.17 |
| Does pretend play | -0.15 | -0.24 | -0.18 | -0.20 |
| Hand or finger mannerisms | 0.11 | 0.20 | 0.48 | † |
| Gauges emotional response to unexpected events from others | -0.10 | † | -0.02 | -0.23 |
| Specific interests | 0.01 | † | 0.17 | † |
| Upset to changes in routine | .001 | -0.43 | † | † |
| Tries to get attention | † | -0.51 | -0.07 | -0.11 |
| Unusual sensory interests | † | 0.27 | 0.04 | 0.39 |
| Plays with multiple toys | † | -0.16 | † | † |
| Repeats the same play actions | † | † | 0.26 | † |
| Has unexpected reactions to sensory experience | † | † | † | 0.01 |
| Attached to unexpected objects | † | † | † | † |

† Indicates not selected in cross-validated elastic net model.

Abbreviations: SLP = Speech Language Pathologist; DT = Developmental Therapist; OT = Occupational Therapist

**Table S2: Putative Thresholds in the Calibration Subsample for the Algorithm**

| **Threshold** | **Accuracy** | **Sensitivity** | **Specificity** | **PPV** | **NPV** |
| --- | --- | --- | --- | --- | --- |
| 0.05 | 0.86 | 1.00 | 0.06 | 0.86 | 1.00 |
| 0.10 | 0.87 | 1.00 | 0.16 | 0.87 | 1.00 |
| 0.15 | 0.90 | 1.00 | 0.31 | 0.89 | 1.00 |
| 0.20 | 0.91 | 0.99 | 0.44 | 0.91 | 0.93 |
| 0.25 | 0.91 | 0.99 | 0.44 | 0.91 | 0.93 |
| 0.30 | 0.92 | 0.99 | 0.50 | 0.92 | 0.94 |
| 0.35 | 0.92 | 0.99 | 0.56 | 0.93 | 0.90 |
| 0.40 | 0.94 | 0.99 | 0.66 | 0.94 | 0.91 |
| 0.45 | 0.94 | 0.98 | 0.75 | 0.96 | 0.86 |
| 0.50 | 0.95 | 0.98 | 0.78 | 0.96 | 0.86 |
| 0.55 | 0.94 | 0.97 | 0.78 | 0.96 | 0.81 |
| 0.60 | 0.93 | 0.95 | 0.81 | 0.97 | 0.74 |
| 0.65 | 0.92 | 0.93 | 0.81 | 0.97 | 0.68 |
| 0.70 | 0.90 | 0.91 | 0.81 | 0.96 | 0.62 |
| 0.75 | 0.89 | 0.90 | 0.84 | 0.97 | 0.60 |
| 0.78 † | 0.88 | 0.88 | 0.84 | 0.97 | 0.56 |
| 0.80 | 0.87 | 0.87 | 0.88 | 0.98 | 0.55 |
| 0.85 | 0.85 | 0.85 | 0.91 | 0.98 | 0.51 |
| 0.90 | 0.82 | 0.81 | 0.91 | 0.98 | 0.45 |
| 0.95 | 0.78 | 0.76 | 0.94 | 0.99 | 0.41 |

† Youden’s J recommended single threshold

Note, for the proposed dual threshold model, we selected 0.50 as a lower threshold and 0.75 as an upper threshold.
